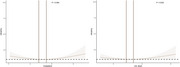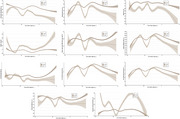# Low Nutritional and Metabolic Abnormalities Predict the Incidence of Alzheimer's Diseases: A Large Prospective Cohort Study

**DOI:** 10.1002/alz70856_097481

**Published:** 2025-12-24

**Authors:** Jiajia Fu, Xueping Chen, Huifang Shang

**Affiliations:** ^1^ West China Hospital, Sichuan University, Chengdu, Sichuan, China

## Abstract

**Background:**

Nutritional and metabolic factors play significant roles in Alzheimer's disease (AD). To date, there have been no large‐scale prospective studies exploring their potential impacts on AD and the changes prior to the onset of AD. This study aimed to comprehensively analyze the temporal changes in body composition and blood nutritional metabolic biomarkers before the onset of AD and to assess whether these factors influence the risk of occurrence of AD using the large UK Biobank database.

**Method:**

Between 2006 and 2010, participants from the UK Biobank who completed body composition assessments and blood tests were included in the analysis. A total of 226,328 participants were enrolled, of whom 1,283 eventually developed AD.

**Result:**

The combination of Cox regression analysis and nested case‐control studies revealed the following findings: 1) Elevated fat percentage, fat mass, and fat‐free mass decreased the risk of AD, and Cholesterol (TC) and Low density lipoprotein cholesterol (LDL‐C) were non‐linearly associated with the occurrence of AD, which both elevated and reduced levels of these lipids were associated with an increased risk of AD (Figure 1). 2) Increases in sex hormone‐binding globulin (SHBG) and aspartate aminotransferase levels increased the risk of AD (HR=1.005, *p* <0.001; HR=1.007(1.003‐1.011, *p* <0.001). 3) Endocrine, nutritional, and metabolic diseases: Diabetes increased the risk of AD (HR=1.307(1.077‐1.586, *p* = 1.007).

Temporal Trends: Before the onset of AD, AD patients exhibited lower weight, vitamin D levels, and body composition compared to their age‐matched controls, while SHBG and aspartate aminotransferase levels were higher (Figure 2).

**Conclusion:**

In summary, the study found that poor nutrition status and metabolic disturbances of the liver and pancreas increased the incidence of AD, potentially serving as earlier biomarkers in the preclinical stage of the disease. Therefore, it is essential to monitor changes in these prodromal biomarkers for different AD.